# Epidemiology of Injuries in Women’s Rugby Sevens: A Systematic Review

**DOI:** 10.3390/sports14020073

**Published:** 2026-02-06

**Authors:** Carlos Braga, Pedro Lopes, Luiz Miguel Santiago, António Cruz-Ferreira

**Affiliations:** 1 Faculdade de Medicina, Universidade de Coimbra, 3000-548 Coimbra, Portugal; pedro.mglopes@hotmail.com (P.L.); luizmiguel.santiago@gmail.com (L.M.S.);; 2 Unidade de Saúde Familiar Norton de Matos, ULS Coimbra, 3030-790 Coimbra, Portugal; 3 Centro de Estudos de Investigação em Saúde, Universidade de Coimbra, 3004-512 Coimbra, Portugal; 4 Federação Portuguesa de Rugby, 1600-131 Lisboa, Portugal; 5 Departamento de Medicina, Instituto do Desporto e Juventude, 1600-190 Lisboa, Portugal

**Keywords:** women’s rugby sevens, epidemiology, athletic injuries, sports medicine, injury prevention

## Abstract

**Background**: Women’s rugby sevens is rapidly expanding, yet injury patterns remain poorly understood, limiting prevention strategies. This systematic review aimed to describe injury incidence, severity, burden, and risk factors across competitive levels. **Methods**: Original studies on senior or U19 women’s rugby sevens reporting ≥ 2 epidemiological variables were included; studies on men, mixed samples without disaggregation, 15-a-side rugby, other sports, or players below U19 were excluded. Searches were conducted in PubMed, Google Scholar, ScienceDirect, SpringerLink, and Scielo (last searched September 2024), supplemented by gray literature and hand searching. Risk of bias was assessed with ROBINS-I, and study quality was assessed with STROBE. Results were tabulated and synthesized narratively due to heterogeneity. **Results**: Fifteen studies were included. Injury incidence ranged from 40.5 to 153.6 per 1000 match h at the elite level and 26.5–46.3 at the community level. Severity was higher in elite players (45.6–124 days) than in community players (29.6–58.4 days). Lower-limb joint/ligament injuries predominated, contact (especially tackling) was the main mechanism, and injuries often occurred in the second half. **Conclusions**: Evidence was limited by small samples, inconsistent reporting, and a moderate risk of bias. Injuries are frequent and severe, especially in elite players, highlighting the need for targeted prevention and improved surveillance.

## 1. Introduction

Rugby sevens is a high-intensity collision sport played by two teams of seven players over two seven-minute halves, with rules like the traditional fifteen-a-side game. Despite its fast-paced nature and global popularity, particularly after its inclusion in the 2016 Olympic Games [1], rugby sevens players engage in more tackles and ball carries into contact compared to rugby union players, presenting a significant injury risk [2,3]. Additionally, rugby sevens features tournament formats where teams play multiple games in a day, resulting in teams with short recovery times and increased physiological stress [3,4].

While injury research in rugby sevens has expanded post-Olympics, most studies focus on male athletes or mix gender data, limiting applicability to women’s rugby sevens [5]. Women’s rugby sevens presents a distinctly high injury risk driven by the combination of repeated high-speed contact, congested tournament schedules, and the specific physiological and biomechanical characteristics of female athletes.

Women face unique injury risks due to physiological and biomechanical differences and variations in match intensity and recovery demands [6,7,8]. Recent research across women’s team sports highlights similarly high injury burdens, reinforcing the need for gender-specific analyses in rugby sevens [9,10,11,12]. Epidemiological studies have shown that female athletes in contact and pivoting sports such as soccer, basketball, and handball have two- to eight-fold higher anterior cruciate ligament (ACL) injury rates compared to their male counterparts [9,10,11,12]. Furthermore, concussion incidence remains consistently higher in women’s team sports, potentially due to sex-based differences in neck strength, hormonal influences, and reporting behaviors [9,10,11,12]. These findings suggest that the injury mechanisms and outcomes seen in women’s rugby sevens are part of a broader trend across female team sports, emphasizing the importance of tailored prevention and load management strategies to reduce injury risk in this population.

In fact, epidemiological evidence demonstrates consistently higher rates of anterior cruciate ligament (ACL) injuries among female athletes, often linked to sex-specific anatomical, hormonal, biomechanical, and neuromuscular factors such as increased knee valgus angles during landing, differences in muscle activation patterns, and fluctuations in ligament laxity across the menstrual cycle [13,14]. Also, sex-based differences in strength, landing mechanics, and fatigue resistance may interact with the demands of rugby sevens to influence both incidence and severity of injuries [13,14].

There is emerging evidence from women’s sevens that injury patterns may diverge from those reported in men’s sevens or women’s fifteens, highlighting soft-tissue injuries (e.g., ligament sprains, muscle strains) and concussion as the most frequently reported injury types [15,16].

To date, no systematic review has exclusively examined injury epidemiology in women’s rugby sevens. Given that rugby sevens is one of the most injury-prone team sports, with incidence rates higher than those in rugby fifteens, a focused investigation into injury patterns is crucial [3,17]. Women’s rugby demonstrated a significantly higher demand for medical attention compared to men’s rugby, as indicated by the greater number of patient encounters, despite both teams having equal roster sizes [18,19]. Given the limited experience of many female athletes in international competition, it is plausible that inexperience could be a contributing risk factor for injury [2,19].

Injury incidence in rugby sevens varies substantially across competitive levels because elite tournaments involve higher match intensity and a greater number of collisions, whereas sub-elite or developmental players may experience increased injury risk due to lower technical proficiency and reduced physical conditioning [20]. In addition, the tournament format in which teams play several matches in a single day creates cumulative fatigue and limited recovery time, which further increases injury likelihood [21].

Conducting this review is, therefore, essential to consolidate the fragmented evidence base, identify consistent patterns, and guide the development of targeted prevention and management strategies for female rugby sevens players.

A clear theoretical framework is also needed to guide the interpretation of injury data in this sport. While incidence reflects how often injuries occur, variables such as severity and burden provide essential insight into how disruptive injuries are for team performance, athlete availability, and resource allocation [3,16]. In a sport with small rosters and congested match schedules, epidemiological measures such as injury severity and injury burden are essential in women’s rugby sevens because they provide insight into how much playing time is lost and how team availability is affected [3,16].

This systematic review aims to analyze epidemiological evidence on injuries in women’s rugby sevens, providing insights into incidence, type, severity, burden, and risk factors to inform injury prevention strategies (Graphical Abstract).

## 2. Materials and Methods

This systematic review was conducted in accordance with the Preferred Reporting Items for Systematic Reviews and Meta-Analyses (PRISMA) 2020 guidelines [22] and was designed using the PICO framework to ensure clarity and focus. This review was not registered in PROSPERO; nevertheless, the review strictly adhered to the PRISMA 2020 guidelines (PRISMA Checklist in Supplementary Materials).and fully documented all methodological procedures to ensure transparency and reproducibility.

In this review, the population included women’s rugby sevens players across all competitive levels, the primary outcomes were epidemiological variables including injury incidence, severity, burden, type, and context, and the ‘intervention’ and ‘comparison’ components were conceptualized as exposure to matches or tournaments under varying conditions, allowing assessment of differences in injury outcomes across competitive levels and tournament formats (Table 1). This framework guided the eligibility criteria and analysis to address the research question: “What are the injury patterns and risks in women’s rugby sevens?” Given that most of the existing literature focuses on male or mixed-gender populations, this study aimed to fill the gap by concentrating specifically on female sevens players. While comparative insights with male sevens and female 15-a-side rugby may be discussed in the interpretation of findings, the primary aim remains to describe and understand injuries within the context of elite women’s sevens rugby.

A comprehensive search of the literature was conducted to identify studies published between 2010 and September 2024 across several databases, including PubMed, Google Scholar, ScienceDirect, SpringerLink, and Scielo. The research was conducted in September 2024. Non-peer-reviewed sources (e.g., World Rugby, Rugby Europe) were included to capture recent, real-world injury data not yet available in peer-reviewed literature, particularly for under-researched groups, like female rugby sevens players. Hand searching was performed by reviewing the reference lists of included articles and relevant systematic reviews to identify additional studies that met the inclusion criteria. Gray literature, such as reports from World Rugby and Rugby Europe, was included to ensure a comprehensive capture of the most current and relevant injury data in women’s rugby sevens, particularly for populations and tournaments that are underrepresented in peer-reviewed publications. Recognizing the potential for variable quality and bias, each gray literature source was critically appraised and triangulated with peer-reviewed evidence to enhance reliability and reduce the risk of publication bias, as recommended in systematic review methodology.

The following search strategy was used: (“rugby sevens” OR “rugby 7 s” OR “women’s rugby sevens” OR “women’s rugby 7 s”) AND (“injury” OR “injuries” OR “athletic injuries” OR athlet* injur* OR “sports injuries” OR “trauma”). For PubMed this search strategy was adapted, resulting in the following search strategy: (“Rugby” [MeSH] OR “Rugby Sevens” OR “Rugby 7 s” OR “Women’s Rugby Sevens” OR “Women’s Rugby 7 s”) AND (“Wounds and Injuries” [MeSH] OR “Athletic Injuries” [MeSH] OR athlet* injur* OR “Trauma”).

Inclusion criteria for retrieved studies were as follows: (1) Original research studies, including prospective and retrospective cohort studies and randomized controlled trials; unpublished and gray literature sources (e.g., organizational reports, surveillance data) were also considered when they provided original epidemiological data relevant to women’s rugby sevens; (2) studies that were specifically focused on 7-a-side women’s rugby seven’s players at the senior level or under-19; (3) studies reporting at least two of the following epidemiological variables: injury incidence, mean severity, type of injury, injury burden, location of the injury, mechanisms of injury, nature of onset, cause of onset of injury, and match period when the injury occurred; (4) publications in English or Portuguese; and (5) studies clearly defining injuries. Studies were eligible for inclusion if they reported at least two of the predefined epidemiological outcomes. During full-text review, study characteristics and outcome data were tabulated and compared against the planned outcome framework to determine whether each study contributed to the synthesis of incidence, severity, burden, or descriptive injury patterns.

Studies were excluded from our review if the included the following: (1) They were focused exclusively on men’s rugby sevens; (2) they presented combined data for both genders without disaggregation; (3) they focused on 15-a-side rugby; (4) they reported injury data from sports other than rugby sevens; (5) they reported data on players below under-19 level; or (6) they were studies that combined data on match and training injuries without separating the two.

**Figure 1:**
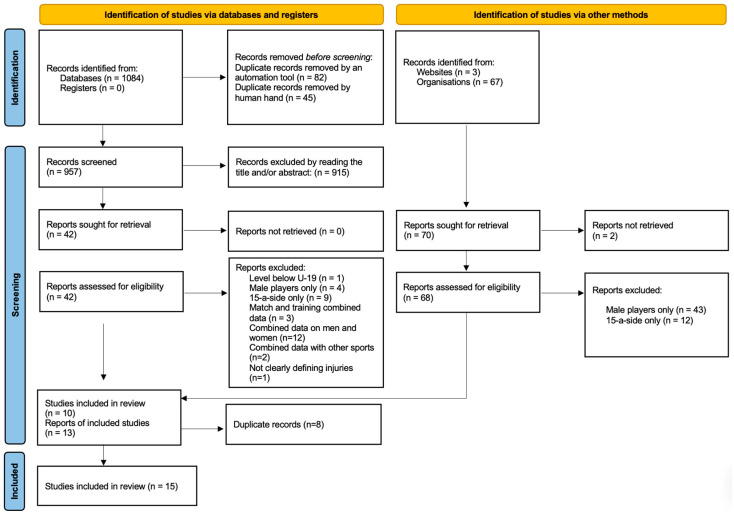
PRISMA literature retrieval and selection.

All included articles were imported into Zotero, and duplicates were removed using Zotero’s automated tools. An additional manual duplicate removal also had to be performed. Two reviewers screened all identified studies based on their titles and abstracts. Studies that did not meet the inclusion criteria were excluded, and at this stage, no detailed justifications for exclusions were documented. Full-text articles were retrieved for the remaining studies and further assessed for eligibility. The details of study selection can be seen in the PRISMA flow diagram (Figure 1). Disagreements between reviewers were resolved through discussion until a consensus was reached.

Data from the included studies were extracted by one reviewer and checked by the other two. During data extraction, any discrepancies between reviewers were discussed and resolved through consensus meetings, and when agreement could not be reached, a third senior reviewer was consulted to ensure the accuracy and consistency of the extracted data. Descriptive variables included were level of play, number of players, tournaments played, and year of play. Data collection was guided by the international consensus statement on injury definitions and data collection procedures in studies of injuries in rugby union [24] (Table 1). Exposure is reported as player match h; incidence rate is reported as number of injuries per 1000 player match h; injury severity is presented in days of absence from competition or practice; injury burden was defined by *Bahr* et al. *(2020)* [23] as the injury incidence rate (per 1000 player match h) multiplied by the mean severity (measured as days lost) of the sample [23]. This provides a comprehensive metric that reflects both how often injuries occur and how serious they are in terms of time lost from sport (Table 1).

For each outcome, the effect measures used in the synthesis included injury incidence expressed as injuries per 1000 player match h, injury severity reported as days lost from participation, and injury burden calculated as incidence multiplied by mean severity; categorical outcomes such as injury type, location, nature, cause, match event, and match period were summarized using proportions and frequencies.

Additional variables sought included participant characteristics (age, competitive level), study characteristics (season or tournament context), and reporting characteristics (funding sources and conflicts of interest). When information was missing or unclear, assumptions were made only when explicitly justified by contextual details provided in the study.

No data imputation or statistical conversions were performed. When studies did not report specific summary statistics, the variable was coded as ‘not reported’ (N/R). Injury incidence, severity, and burden values were extracted in the units provided by each study.

Defining the variable that is not taken into consideration in the consensus statement is equally crucial: the level of competition. The classification system used in this review stratifies studies into three levels to ensure comparability and to contextualize findings. We decided to consider “level one” as any competition regarding international elite tournaments (World Cup, Olympics, Sevens series, Challenger series, and European tournaments). “Level two” was classified as any domestic elite competition (Premier 15 s (UK), Super W (Australia), or France’s Elite 1). “Level three” included all community tournaments (university leagues, regional club competitions, or local amateur sevens events). When the original studies used different or unclear definitions, these were standardized according to the outlined classification criteria, and discrepancies were resolved through discussion between two reviewers to ensure consistency and comparability across studies. A narrative synthesis of the included studies was performed, summarizing key outcomes such as injury incidence, severity, and risk factors.

Given the heterogeneity of study designs and outcome reporting, a narrative synthesis approach was used, whereby results were grouped and compared descriptively according to key epidemiological variables (incidence, severity, burden, injury type, mechanism, and context). Studies were examined for patterns and consistencies across competitive levels and injury characteristics. No meta-analysis was performed, and therefore, no statistical models or software for heterogeneity assessment were required. No formal methods were used to explore heterogeneity. Variability across studies was assessed qualitatively based on differences in competitive level, study design, and injury definitions. No sensitivity analyses were conducted, as the heterogeneity of study designs and outcome measures precluded quantitative assessment of robustness.

Extracted data from individual studies were organized and tabulated in structured summary tables displaying study characteristics and injury outcomes, and results were visually presented using tables and the PRISMA flow diagram to illustrate study selection and data synthesis.

The quality of the included studies was assessed using the STROBE (Strengthening the Reporting of Observational Studies in Epidemiology) checklist. Studies were rated as follows: “High quality” if they fulfilled more than 80% of the STROBE criteria; “Moderate quality” if they met between 50 and 80% of the criteria; and “Low quality” if they met less than 50% of the criteria. On the other hand, the risk of bias for each study was assessed using the ROBINS-I (Risk of Bias in Non-Randomized Studies of Interventions) tool. Each domain was rated as low, moderate, serious, or critical risk of bias, and an overall risk of bias was provided for each study. To enhance transparency, the complete results of both the STROBE quality assessment and the ROBINS-I risk-of-bias evaluation for all included studies are presented in Table 2 and Table 3. Both assessments were independently conducted by two reviewers to ensure methodological rigor and consistency. Disagreements in the quality and risk-of-bias assessment were resolved by consensus among the reviewers.

## 3. Results

From 1084 database records and 70 additional sources, 957 records were screened. After exclusions for duplicates, irrelevance, and ineligible study characteristics (e.g., male-only samples, combined data, 15-a-side rugby), 15 studies met the inclusion criteria for the systematic review (Figure 1). There were no Portuguese studies in this search. No studies that initially appeared eligible were later excluded after full-text review.

With a few minor variations, all of the articles chosen for this systematic review offered precise definitions of injuries that were essentially consistent with the global consensus statement on injury definitions and data collection procedures in studies of injuries in rugby union. Ref. [3] Studies defined injury as “*any physical complaint caused by transfer of energy that exceeded the body’s ability to maintain its structural and/or functional integrity, sustained by a player during a rugby match”* [23,24]. Fuller et al. [25] chose to define injury as “*Any injury sustained during a Sevens World Series Tournament match or training activity that prevents a player from taking a full part in all normal training activities and/or match play for more than one day following the day of injury”*. This definition was consistently applied across multiple competitions, including the Sevens World Series, 2020 Olympics, 2022 and 2024 Challenger Series, SVNS tournaments, and Rugby Europe 7s competitions [1,19,26,27,28,29,30].

From the fifteen studies included, nine [1,19,26,27,28,29,30,31,32] focused on level one players, covering the Challenger Series, Sevens Series, World Cup, and Olympics. The remaining six [5,33,34,35,36,37] examined level three competitions, primarily tracking tournaments in the USA. The reported total exposure for players ranged between 64.0 player match h for the 2014 World Cup tournament and 6285.1 player match h, as reported by *Ma and Lopez* in their level three contact and non-contact studies [5,33,34,35].

**Table 2:** Data extracted from level one studies included in this review.

Author	Fuller and Taylor [1]	Fuller et al. [19]	Fuller and Taylor [26]	Fuller and Taylor [27]	Fuller and Taylor [28]	Fuller and Taylor [29]	Rugby Europe [30]			Bailey [31]	Fuller and Taylor [32]
Level	One	One	One	One	One	One	One	One	One	One	One
Tournaments	CHLRS	Olympics	Olympics	CHLRS	SVNS	RWCS	Championship	Trophy	Conference	SVNS; European Grand Prix	SVNS
Year	2022	2016	2020	2024	2011–23	2022	2022–23	2022–23	2022–23	2018–19	2023–24
Quality	Moderate	High	High	Moderate	High	High	Moderate	Moderate	Moderate	Moderate	High
Overall risk of bias	Moderate	Moderate	Moderate	Moderate	Moderate	Moderate	Moderate	Moderate	Moderate	Moderate	Moderate
Number of players	N/R	N/R	N/R	N/R	N/R	N/R	N/R	N/R	N/R	25	N/R
Number of injuries	9	8	11	28	551	9	16	9	6	11	72
Total exposure (player/h)	93.1	112.5	101.3	298.9	5351.0	84.9	68	68	26	71.6	797.1
Incidence rate (per 1000 player/h, 95% CI)	97 (50–186)	71.1 (35.6–142.2)	109 (60–196)	93.7 (64.7–135.7)	103.0 (94.7–111.9)	106.0 (55.2–203.7)	72.0 (38.0–106.0)	40.5 (14.6–66.5)	70.7 (16.2–125.2)	153.6 (62.8–244.4)	90.3 (71.7–113.8)
Mean severity (days absent from sport)	63 (5–120)	92.0 (22.5–161.5)	124 (61–187)	104.8 (49.7–159.8)	51.5 (45.9–57.6)	52.7 (12.5–92.8)	N/R	N/R	N/R	45.6 (0.0–108.6)	81.0 (55.2–106.8)
Injury burden (days absence/1000 player match h)	N/R	N/R	N/R	9813	N/R	N/R	N/R	N/R	N/R	7011.2 (6397.8–7624.5)	7316 (5807–9217)
Type (%, 95% CI)											
Bone	37.5 (4.0–71.0)	12.5 (0–35.4)	18.2 (0–41.0)	17.9 (3.7–32.0)	12.7 (9.9–15.5)	11.1 (0–31.6)	17.8 (0.0–36.1)	(–)	16.7 (0.0–46.5)	9.6 (7.0–12.1)	6.9 (1.1–12.8)
Joint/ligament	37.5 (4.0–71.0)	62.5 (29.0–96.0)	54.5 (25.1–84.0)	46.4 (28.0–64.9)	42.7 (38.6–46.9)	22.2 (0–49.4)	37.5 (13.8–61.2)	55.5 (23.0–88.0)	16.7 (0.0–46.5)	80.9 (77.4–84.3)	50.0 (38.5–61.5)
Muscle/tendon	12.5 (0–35.4)	12.5 (0–35.4)	18.2 (0–41.0)	14.3 (1.3–27.2)	23.8 (20.3–27.4)	11.1 (0–31.6)	12.5 (0.0–28.7)	22.2 (0.0–49.4)	(–)	5.4 (3.4–7.4)	22.2 (12.6–31.8)
Skin	12.5 (0–35.4)	(–)	(–)	(–)	1.8 (0.7–2.9)	(–)	12.5 (0.0–28.7)	(–)	16.7 (0.0–46.5)	(–)	1.4 (0–4.1)
CNS/PNS	(–)	12.5 (0–35.4)	9.1 (0–26.1)	21.4 (6.2–36.6)	17.8 (14.6–21.0)	22.2 (0–49.4)	17.8 (0.0–36.1)	22.2 (0.0–49.4)	33.3 (0.0–71.0)	4.2 (2.4–5.9)	15.3 (7.0–23.6)
Other	(–)	(–)	(–)	(–)	(–)	33.3 (2.5–64.1)	(–)	(–)	16.7 (0.0–46.5)	(–)	4.2 (0–8.8)
Location (%, 95% CI)											
Head/neck	25.0 (0–55.0)	12.5 (0–35.4)	9.1 (0–26.1)	25.0 (9.0–41.0)	24.4 (20.8–28.0)	33.3 (2.5–64.1)	31.3 (8.6–54.0)	22.2 (0.0–49.4)	66.6 (28.9–10.0)	4.2 (2.4–5.9)	22.2 (12.6–31.8)
Upper limb	25.0 (0–55.0)	25.0 (0–55.0)	45.5 (16.0–74.9)	21.4 (6.2–36.6)	18.4 (15.1–21.6)	(–)	18.8 (0.0–37.9)	55.6 (23.1–88.1)	16.7 (0.0–46.5)	12.0 (9.1–14.8)	9.7 (2.9–16.6)
Trunk	(–)	(–)	(–)	3.6 (0–10.4)	6.4 (4.3–8.4)	11.1 (0–31.6)	(–)	11.1 (0.0–31.6)	(–)	(–)	2.8 (0–6.6)
Lower limb	50.0 (15.4–84.6)	62.5 (29.0–96.0)	45.5 (16.0–74.9)	50.0 (31.5–68.5)	50.9 (46.7–55.1)	55.6 (23.1–88.0)	50.0 (25.5–74.5)	22.2 (0.0–49.4)	16.7 (0.0–46.5)	83.9 (80.7–87.1)	65.3 (54.3–76.3)
Other	(–)	(–)	(–)	(–)	(–)	(–)	(–)	(–)	(–)	(–)	(–)
Mechanism of Injury (%, 95% CI)											
Contact	N/R	83.3 (53.5–100)	50.0 (19.0–81.0)	78.6 (63.4–93.8)	86.9 (84.0–89.8)	88.9 (68.4–100)	87.5 (71.3–100.0)	88.9 (68.4–100.0)	66.7 (29.0–100.0)	N/R	73.6 (63.4–83.8)
No-contact	N/R	16.7 (0–46.5)	50.0 (19.0–79.5)	21.4 (6.2–36.6)	12.3 (4.3–20.3)	11.1 (0–31.6)	12.5 (0.0–28.7)	11.1 (0.0–31.6)	33.3 (0.0–71.0)	N/R	26.4 (16.2–36.6)
Match event (%, 95% CI)											
Collision	N/R	N/R	N/R	7.1 (0–16.7)	14.6 (11.6–17.6)	N/R	12.5 (0.0–28.7)	(–)	(–)	N/R	16.4 (7.5–25.3)
Kicking	N/R	N/R	N/R	(–)	0.8 (0.0–1.5)	N/R	(–)	(–)	(–)	N/R	1.5 (0–4.4)
Lineout	N/R	N/R	N/R	(–)	0.6 (0–1.2)	N/R	(–)	(–)	16.7 (0.0–46.5)	N/R	(–)
Maul	N/R	N/R	N/R	(–)	0.0 (-)	N/R	(–)			N/R	(–)
Ruck	N/R	N/R	N/R	3.6 (0–10.4)	7.8 (5.5–10.0)	N/R	6.3 (0.0–18.2)	11.1 (0.0–31.6)	16.7 (0.0–46.5)]	N/R	3.0 (0–7.1)
Running	N/R	N/R	N/R	17.9 (3.7–32.0)	10.0 (7.5–12.6)	N/R	(–)	(–)	(–)	N/R	16.4 (7.5–25.3)
Scrum	N/R	N/R	N/R	(–)	0.4 (0–0.9)	N/R	(–)	(–)	(–)	N/R	(–)
Tackled	N/R	N/R	N/R	42.9 (24.5–61.2)	35.8 (31.7–39.9)	N/R	31.3 (8.7–54.3)	33.3 (2.5–64.1)	33.3 (0.0–71.0)	N/R	38.8 (27.1–50.5)
Tackling	N/R	N/R	N/R	21.4 (6.2–36.6)	25.2 (21.5–28.9)	N/R	37.5 (13.8–61.2)	44.4 (11.9–76.9)	(–)	N/R	14.9 (6.4–23.5)
Other	N/R	N/R	N/R	7.1 (0–16.7)	4.9 (3.1–6.8)	N/R	(–)	(–)	(–)	N/R	9.0 (2.1–15.8)
Nature (%, 95% CI)											
Acute	100.0 (–)	87.5 (64.6–100)	90.9 (73.9–100)	92.9 (83.3–100)	93.5 (91.4–95.5)	100.0 (–)	93.7 (81.8–100.0)	88.9 (68.4–100.0)	100 (–)	N/R	84.7 (76.4–93.0)
Gradual	0.0 (–)	12.5 (0–35.4)	9.1 (0–26.1)	7.1 (0–16.7)	6.5 (4.5–8.6)	0.0 (–)	6.3 (0.0–18.2)	11.1 (0.0–31.6)	(–)	N/R	15.3 (7.0–23.6)
Match period (%, 95% CI)											
First half	55.6	N/R	36.4 (7.9–64.8)	50.0 (31.5–68.5)	41.3 (37.1–45.4)	44.4 (12.0–76.9)	37.5 (13.8–61.2)	11.1 (0.0–31.6)	50.0 (10.0–90.0)	N/R	36.1 (25.0–47.2)
Second half	33.3	N/R	63.6 (35.2–92.1)	50.0 (31.5–68.5)	58.7 (54.6–62.9)	55.6 (23.1–88.0)	62.5 (38.8–86.2)	78.8 (52.1–100.0)	33.3 (0.0–71.0)	N/R	63.9 (52.8–75.0)
Other	11.1	(–)	(–)	(–)	(–)	(–)	(–)	11.1 (0.0–31.6)	16.7 (0.0–46.5)	(–)	(–)

CHLRS: Challenger Series; CI: Confidence interval; CNS/PNS: Central Nervous System/Peripheral Nervous System; N/R: Not reported; SVNS: Sevens Series; RWCS: Rugby World Cup Sevens.

**Table 3:** Data extracted from level three studies included in this review.

Author	Lopez et al. [34]	Ma et al. [33]	Lopez et al. [5]	Ma et al. [35]	Borthwick et al. [36]	Victoria et al. [37]
Level	Three	Three	Three	Three	Three	Three
Tournaments	USARST	USARST	USARST	USARST	USARST	USARST
Year	2010–2013	2010–2013	2010–2014	2010–2015	2011–2016	2010–2015
Quality	High	Moderate	Moderate	Moderate	Moderate	Moderate
Overall risk of bias	Moderate	Moderate	Moderate	Moderate	Moderate	Moderate
Number of players	3876	3876	61	10,328	N/R	10,328
Number of injuries	120	173	67	167	95	523
Total exposure (player/h)	2590.8	2590.8	454.0	6285.1	N/R	N/R
Incidence rate (per 1000 player/h, 95% CI)	8.1	46.3 (38.4–55.4)	85.9	26.5 (22.7–30.9)	12.9	59.0
Mean severity (days absent from sport)	36.7 (20.8–52.6)	45.9 (33.1–58.7)	29.6	58.4	N/R	56.7
Injury Burden	N/R	N/R	N/R	N/R	N/R	N/R
**Type (%, 95% CI)**						
Bone	(–)	14.0	11.1 (2.4–38.9)	2.99	5.0	N/R
Joint/ligament	(–)	36.0	38.9 (18.2–64.5)	40.12	84.0	N/R
Muscle/tendon	(–)	(–)	27.8 (10.9–54.7)	31.14	(–)	N/R
Skin	(–)	(–)	0.0 (–)	11.38	(–)	N/R
CNS/PNS	100.0	13.0	22.2 (7.7–49.5)	5.99	(–)	N/R
Other	(–)	(–)	0.0 (–)	5.39	(–)	N/R
Location (%, 95% CI)						
Head/neck	100.0	18.0	27.8 (10.9–54.7)	8.98	(–)	N/R
Upper limb	(–)	26.0	33.3 (14.4–59.7)	2.99	(–)	N/R
Trunk	(–)	8.0	0.0 (–)	19.16	(–)	N/R
Lower limb	(–)	44.0	38.9 (18.2–64.5)	61.08	100.0	N/R
Other	(–)	4	(–)	3.59	(–)	N/R
Mechanism of Injury (%, 95% CI)						
Contact	100.0	87.1	N/R	(–)	69.0	100
No-contact	(–)	11.9	N/R	100.0	31.0	(–)
Match event (%, 95% CI)						
Collision	33.3 (13.1–53.5)	19.3	N/R	(–)	(–)	(–)
Kicking	(–)	-	N/R	(–)	(–)	(–)
Lineout	(–)	-	N/R	(–)	(–)	(–)
Maul	(–)	-	N/R	0.6	(–)	(–)
Ruck	(–)	4.6	N/R	2.4	(–)	(–)
Running	(–)	20.4	N/R	38.9	31.0	(–)
Scrum	(–)	1.9	N/R	1.2	(–)	(–)
Tackled	19.0 (2.2–35.8)	40.9	N/R	35.3	60.0	82.0
Tackling	42.9 (21.7–64.1)	31.3	N/R	21.4 (6.2–36.6)	25.2 (21.5–28.9)	N/R
Other	4.8 (0–13.9)	(–)	N/R	21.6	(–)	18.0
Nature (%, 95% CI)						
Acute	100.0 (–)	95.0	100.0	90.0	N/R	100.0
Gradual	(–)	5.0	0.0	10.0	N/R	(–)
Match period (%, 95% CI)						
First half	46.0	41.3 (37.1–45.4)	N/R	N/R	N/R	44.4 (12.0–76.9)
Second half	54.0	58.7 (54.6–62.9)	N/R	N/R	N/R	55.6 (23.1–88.0)
Other	(–)	(–)	(–)	N/R	N/R	(–)

CI: Confidence interval; CNS/PNS: Central Nervous System/Peripheral Nervous System; N/R: Not reported.

### 3.1. Injury Incidence

Nine studies included in this review provided information about the overall match injury incidence rate for level one rugby sevens tournaments (Table 2), while two provided data for level three (Table 3). This distinction between levels is important, as differences in match intensity, player conditioning, and medical support may influence injury risk. Additionally, one study [34] provided the match injury incidence of concussion for level three, while another [36] presented the injury incidence rate of ankle injuries for the same level. Some studies focused specifically on contact or non-contact injuries, which may account for variations in the reported rates (Table 2 and Table 3). At level three, one study [34] only reported the contact injury incidence, while another [33] focused on non-contact injuries (Table 3). Overall, injury incidence at level one ranged from 40.5 (95% CI 14.6–66.5) per 1000 player match h [30] to 153.6 per 1000 player match h (95% CI 62.8–244.4) [31]. For level three, the overall injury incidence varied from 26.5 (95% CI 22.7–30.9) [35] per 1000 player match h to 46.3 (95% CI 38.4–55.4) [33] per 1000 player match h.

### 3.2. Injury Severity

Only one [30] study did not supply data on the severity of injuries involving level one players. The mean severity of injuries for level one players ranged between 45.6 days (95% CI 0.0–108.6) [31] and 124 days (95% CI 61–187) [26]. The mean severity for level three ranged from 29.6 days [5] to 45.9 (95% CI 33.1–58.7) [33]. Regarding non-contact injuries among level three players, the mean severity was 58.4 days [35], and in contact injuries, it was 56.7 days [37]. In level three competitions, the higher mean severity of non-contact injuries was primarily associated with lower-limb soft-tissue injuries such as hamstring strains, quadriceps strains, and calf muscle tears, which were the most frequently reported non-contact injury types across the included studies. Concussive injuries caused a mean severity of 36.7 days (95% CI 20.8–52.6) in level three players [34].

### 3.3. Injury Burden

Injury burden in level one competitions ranged between 7011.2 days absence/1000 player match h (95% CI 6397.8–7624.5) [31] and 9813 days-absence/1000 player-match h [27]. No data were available regarding level three players (Table 3).

### 3.4. Injury Type and Location

All the studies with level one players provided information on the type of injuries. Aligning with international consensus definitions [24], joint/ligament injuries were the most frequent type of injuries in the majority of the studies regarding level one players that presented this type of data, ranging from 16.7% (95% CI 0.0–46.5) [30] to 80.9% (95% CI 77.4–84.3) [31]. Muscle/tendon, Central Nervous System/Peripheral Nervous System (CNS/PNS), and bone injuries tied for the second most frequent type of injury, with their proportions ranging from 5.4% (95% CI 3.4–7.4) [31] to 23.8% (95% CI 20.3–27.4) [28], 4.2% (95% CI 2.4–5.9) [31] to 33.3% (95% CI 0.0–71.0) [30], and 6.9% (95% CI 1.1–12.8) [34] to 37.5% (95% CI 4.0–71.0) [1], respectively. For level three, all studies provided data, with joint/ligament injuries as the most common, ranging from 36.0% [33] to 84.0% [36] (Table 3). Only one [37] of the fifteen articles did not report injury locations. Four main injury locations were identified in 86% of the articles: head/neck, upper limb, trunk, and lower limb. The remaining percentage added a fifth location categorized as “Other”. At level one, lower limb injuries were the most frequent, ranging between 16.7% (0.0–46.5) [30] and 83.9% (95% CI 80.7–87.1) [31], while trunk injuries were the least common, with their proportion ranging from 0.0% [1,19,26] to 11.1% (95% CI 0–31.6) [29,30] (Table 1). On level three players, the lower limb was also the most injured region of the body, ranging from 38.9% (95% CI 18.2–64.5)^18^ to 100% [36] (Table 3).

### 3.5. Match Event

Nine studies [28,29,30,32,33,34,35,36,37] provided information about the match event leading to injury. The tackle was the event of the game where the majority of injuries occurred at both level one (53.7% [28] to 64.6% [30]) and level three competitions (35.3% [35] to 82.0% [37]) (Table 3). However, at level one rugby tournaments, being tackled was more often linked to injuries (31.3 [95% CI 8.7–54.3] [30] to 42.9% [95% CI 24.5–61.2] [27]) than tackling (14.9% [95% CI 6.4–23.5 ] [28] to 44.4% [95% CI 11.9–76.9] [30]). On the other hand, for concussive injuries in level three players, tackling was the most frequently associated event (42.9% [95% CI 21.7–64.1]), followed by collision (33.3% [95% CI 13.1–53.5]) [34].

### 3.6. Mechanism of Injury

Three studies did not supply information about this metric [1,5,31]. On both levels, contact was most often the primary cause of injury onset, compared to non-contact injuries, with overall percentages ranging from 50.0% (95% CI 19.0–81.0) [26] to 88.9% (95% CI 68.4–100) [29]. Among the twelve studies that provided data, injuries were more frequently acute in both levels, ranging from 87.5 (95% CI 64.6–100) [19] to 100% [1,5,29].

### 3.7. Match Period

Ten studies reported the match period when the injury occurred. In five of these, level one injuries were more frequent in the second half (Table 2 and Table 3). In the 2022 Challenger Series article by *Fuller* et al., 55.6% of the injuries occurred in the first half [1], while in the 2024 Challenger Series by *Fuller* et al., injuries were evenly distributed [27]. Regarding level three, concussive injuries occurred 54.0% in the second half [34].

## 4. Discussion

Total match exposure varied notably across studies, reflecting differences in data collection periods (e.g., single events vs. full seasons). While this limits direct comparison, incidence rates offer a more reliable metric by adjusting for exposure. Reported incidences ranged from 46.3 per 1000 player match h at the community level [33] to 153.6 at the elite international level [31], with higher values consistently observed in top-tier competitions. This gradient suggests that greater physical demands, intensified match intensity, and congested schedules at elite tournaments may elevate injury risk [19,38]. Such patterns reinforce the need for level-specific surveillance systems and targeted prevention strategies.

Importantly, elite women’s rugby sevens demonstrated higher injury incidence than women’s rugby union (15-a-side) [17], aligning with previous evidence in men’s rugby sevens [27]. The small squad size, faster pace, and higher tackle frequency likely contribute to this elevated risk. These findings underscore rugby sevens as a uniquely high-risk format, where interventions addressing tackle technique, player workload management, and tournament scheduling may be critical to reducing injury burden and sustaining health [17].

At the elite level, injuries in women’s rugby sevens [31] tend to be more severe than those reported in men’s rugby sevens [3]. Recent research suggests that hormonal fluctuations may influence muscle protein synthesis, neuromuscular control, and recovery times, potentially affecting injury outcomes in female athletes [7]. In the context of rugby sevens, where players are exposed to repeated high-intensity collisions and short recovery periods, these fluctuations may contribute to both a higher risk of soft-tissue injuries and prolonged recovery following time-loss injuries. Hormonal influences on tissue healing and fatigue resistance may, therefore, represent a biological factor that helps explain these patterns [7]. Integrating this line of research underscores the importance of considering female-specific physiology when interpreting injury incidence and developing tailored prevention and recovery strategies.

At the community level, injuries were generally less severe, likely reflecting lower match intensity, while both contact and non-contact injuries contributed substantially to time loss. Although the small difference between them may not be clinically significant, the extended recovery periods highlight the serious impact injuries have on players and teams. Prolonged absences may affect squad depth and preparation for major tournaments, and may influence long-term player development and retention in the sport. Compared to women’s 15-a-side rugby [25], severity in rugby sevens can be comparable or even higher, reinforcing the sport’s faster pace and increased high-intensity actions as contributors to more severe injuries [2,3,17].

This systematic review reveals a notably high injury burden in elite women’s rugby sevens, ranging from 7011.2 days (95% CI 6397.8–7624.5) [31] to 9813 days of absence per 1000 player match h [27]. Compared to men’s rugby sevens [26,27,29,32] and women’s 15-a-side rugby [25,29], the burden in women’s sevens is considerably higher. Injury burden is a standardized metric that integrates both incidence and severity, expressed as the total number of days lost per 1000 player match h. However, when derived from small numbers of injuries, the metric can be disproportionately influenced by a few severe cases with long recovery periods, resulting in inflated values.

In elite women’s rugby sevens, joint/ligament injuries were the most frequent, with proportions ranging from 22.2% (95% CI 0–49.4) [29] to 80.9% (95% CI 77.4–84.3) [31], which is similar to patterns observed in men’s rugby sevens and women’s 15-a-side rugby union,; these injuries are also the most common. Muscle/tendon, CNS/PNS, and bone injuries were also prevalent, with their proportions varying considerably across studies, suggesting that game dynamics, player roles, and physiological factors influence injury risk. This contrasts with men’s sevens, where joint/ligament and muscle/tendon were responsible for over two-thirds of the injuries reported [3]. This finding should raise awareness for bone and CNS/PNS injuries, in order to develop strategies and mechanisms to decrease these types of injuries. At the community level (level three), joint/ligament remained the most frequent type of injury, ranging from 36.0% [33] to 38.9% [5], differing from men’s sevens at the same level of play, where muscle/tendon occupied the first position on the most common type of lesions. These results emphasize the need for tailored injury prevention strategies in women’s rugby sevens, particularly targeting joint and ligament vulnerabilities. Evidence from the rugby union demonstrates that structured neuromuscular warm-up and injury prevention programs, such as the World Rugby Activate program, are effective for reducing the incidence of lower-extremity injuries, especially anterior cruciate ligament (ACL) and ankle sprains [39]. Incorporating such validated programs into women’s rugby sevens may help reduce both injury risk and long-term player absence.

The lower limb was the most common injury location in women’s rugby sevens at both the elite (level one) and community (level three) levels, agreeing with men’s sevens and women’s 15-a-side rugby union at an international elite level [3,25]. This highlights the need for injury prevention strategies targeting biomechanical demands on players’ lower limbs. On the other hand, at the community level, this pattern differs from men’s sevens, where head/neck injuries are more frequent [3]. The finding of 100% lower limb injuries in one level three study [36] may be attributed to its specific focus on ankle injuries, which likely influenced the reported distribution.

Consistent with findings in men’s rugby sevens and women’s 15-a-side rugby union [3,25], most injuries at level three and level one competitions resulted from contact events, with their proportions ranging from 50.0% [26] to 88.9% [29] for level one and 87.1% at the community level [33]. The tackle event was the most common event leading to injury, followed by running and collision. At the elite level, 53.7% [18] to 64.6% [30] occurred during tackles, with being tackled leading more often to injuries than tackling itself, which is similar to men’s sevens and women’s 15-a-side rugby. These differences might highlight the increased vulnerability of the player being tackled, likely due to the inability to control the event intensity and angle, elevating the risk of injury, but more research is needed to confirm this statement. Regarding concussive injuries in community-level competitions, the dynamics shifted, with tackling being more associated with injuries (42.9% (95% CI 21.7–64.1) [34] than being tackled. Collision was the second most frequent event leading to concussion, indicating that direct impacts, irrespective of the play phase, are critical areas for injury prevention. Given the high prevalence of tackle-related injuries identified in this review, medical staff should be aware of concussion-management guidelines. The Consensus Statement on Concussion in Sport from the 6th International Conference on Concussion in Sport–Amsterdam 2022 provides clear, evidence-based recommendations for recognition, management, and return-to-sport decision-making [40]. The findings of this systematic review reinforce the importance of strict adherence to these protocols. Moreover, they emphasize the need for further evaluation of how effectively such measures are being implemented in the women’s game, where limited resources and fewer medical personnel may challenge optimal delivery.

Recent changes to tackle laws have been implemented as part of efforts to reduce high-contact collisions and associated injuries, particularly concussions. The World Rugby Lower Tackle Height Law Trial, conducted in several countries, demonstrated that reducing the legal tackle height to the base of the sternum significantly decreased head-to-head contact [41]. Our findings of a high proportion of tackle-related injuries support the relevance of interventions such as law modifications and technique-based education.

Regarding ankle injuries at the community level, 60% of these injuries also resulted from tackle events, while 31% occurred during running [36]. Coaching strategies should also prioritize proper tackling techniques and safe playing practices, while enhancing player conditioning and awareness of tackle-related risks, particularly at the elite level, to help reduce injury incidence [39,40].

Injuries occurred more frequently in the second half of matches, suggesting that fatigue may be a contributing factor. However, current evidence is insufficient to draw a definitive conclusion. Future studies should report injury timing in smaller intervals (e.g., by quarter or 5 min periods) to allow for more accurate assessments.

At the elite level, testing modified competition models, with fewer matches per day and longer rest intervals, could help reduce injury rates and create a safer environment for female athletes. Evidence from rugby and other sports supports this rationale [17,38]. Providing additional recovery time may reduce cumulative fatigue and lower the risk of overuse and non-contact injuries, thereby creating a safer competition structure for female athletes [17,38]. Importantly, while the available studies describe the timing of injuries within the match (first vs. second half), they do not provide information about the broader competitive context. Specifically, none of the included studies report how many matches players completed on the same day or in which match of the tournament the injury occurred. Future research should, therefore, detail the competition schedule, including the number of matches per day and, when injuries occur, the specific match in which they took place.

A key strength of this review is the rigorous assessment of study quality and risk of bias with validated tools, which strengthened the interpretation of findings. Some aspects of the available evidence warrant consideration when interpreting these findings. Data were not available for level two competitions, leaving a gap in understanding the full spectrum of play. In a few studies, exposure from elite (level 1) and community (level 3) settings was reported together, which may have influenced variability in injury rates. Two-thirds of level one data (six of nine studies) came from World Rugby reports produced by the same research group, which could limit the diversity of perspectives. Furthermore, many studies compared women’s data with male rugby cohorts, which, while useful for context, may reduce focus on the specific characteristics of women’s rugby sevens. In addition, differences in injury definitions, incomplete reporting of confidence intervals, and variations in sample size may have affected comparability across studies. Review processes may also have introduced limitations. Although multiple databases and gray literature sources were searched, relevant studies published in other languages or those not indexed may have been missed. The heterogeneity of study designs precluded meta-analysis, requiring narrative synthesis, which may increase subjectivity. Risk-of-bias assessment and data extraction were performed manually, which may introduce reviewer bias despite standardized methods. Alternative analytical frameworks and the inclusion of additional databases could have expanded the range of eligible studies and enhanced the diversity of the evidence base. Future research should consider integrating these broader search strategies and exploring complementary methodological tools.

Overall, these findings have important implications for practice, policy, and future research. Implementing structured neuromuscular warm-up programs, tackle-technique training, and strict concussion-management protocols may help reduce injury risk in women’s rugby sevens. Policy initiatives, such as lowering tackle height, enhancing match-scheduling guidelines, and strengthening surveillance systems, could further improve player safety. Future research should focus on filling gaps at intermediate competition levels, improving standardization of injury reporting, and examining female-specific physiological factors, such as hormonal influences, that may partly explain injury patterns in this population.

## 5. Conclusions

This systematic review provides the first comprehensive analysis of injury epidemiology in women’s rugby sevens across elite (level one) and community (level three) competitions. Findings highlight a high injury incidence, particularly at the elite level, with joint/ligament injuries in the lower limb being most common.

These findings underscore the unique injury risks faced by women rugby athletes and highlight the urgent need for gender-specific injury prevention and management strategies. Enhanced recovery protocols, refined competition scheduling, and improved tackling techniques should be prioritized to reduce injury burden and support the long-term health of female players. Future research should also investigate injury patterns in domestic elite competitions (level two) to build a more complete understanding of injury risks and better inform care and policy for women across all levels of rugby sevens.

## Figures and Tables

**Table 1:** Variables.

Variable	Category	Operational Definition
Level of Play	Descriptive	The competitive level of the participants (e.g., youth, elite, and international).
Number of Players	Descriptive	Total number of athletes included in the dataset or study.
Tournaments Played	Descriptive	Number of distinct tournaments or events participated in during the study period.
Year of Play	Descriptive	Calendar year(s) in which the data were collected.
Match Exposure	Epidemiological	Total player match h (1 player playing for 1 h = 1 player-h).
Injury Incidence Rate	Epidemiological	Number of injuries per 1000 player match h.
Mean Injury Severity	Epidemiological	Average number of days lost of training or competition due to injury.
Injury Burden	Epidemiological	Product of injury incidence rate and mean injury severity, as defined by Bahr et al. (2020). [23]
Type of Injury	Epidemiological	Clinical classification (e.g., sprain, strain, fracture, and concussion).
Location of Injury	Epidemiological	Anatomical site of injury (e.g., knee, ankle, and head).
Nature of Onset of Injury	Epidemiological	Classification as acute (sudden) or gradual (overuse).
Cause of Onset of Injury	Epidemiological	Mechanism leading to injury (e.g., contact, non-contact, and overexertion).
Match Event Leading to Injury	Epidemiological	Specific phase of play or action (e.g., tackle, scrum, and open play) during which the injury occurred.
Match Period of Injury	Epidemiological	Time during the match when the injury occurred (e.g., 1st half, 2nd half, and extra time).

## Data Availability

The data presented in this study are available on request from the corresponding author due to privacy and ethical restrictions.

## Supplementary Materials

The following supporting information can be downloaded at https://www.mdpi.com/article/10.3390/sports14020073/s1: PRISMA Checklist.

## Abbreviations

The following abbreviations are used in this manuscript:

ACL	Anterior cruciate ligament
CHLRS	Challenger Series
CI	Confidence interval
CNS	Central Nervous System
N/R	Not reported
PICO	Population, Intervention, Comparison, and Outcome
PNS	Peripheral Nervous System
PRISMA	Preferred Reporting Items for Systematic Reviews and Meta-Analyses
RWCS	Rugby World Cup Sevens
SVNS	Sevens Series
